# Rhizosphere Microbial Communities and Heavy Metals

**DOI:** 10.3390/microorganisms9071462

**Published:** 2021-07-08

**Authors:** Anna Barra Caracciolo, Valentina Terenzi

**Affiliations:** Water Research Institute, National Research Council, 00010 Rome, Italy; terenzi.1799155@studenti.uniroma1.it

**Keywords:** plants, prokaryotic communities, microbiome, chemical dialogue, exudates, secondary metabolites, stress response, holobiont, hologenome, metaorganism

## Abstract

The rhizosphere is a microhabitat where there is an intense chemical dialogue between plants and microorganisms. The two coexist and develop synergistic actions, which can promote plants’ functions and productivity, but also their capacity to respond to stress conditions, including heavy metal (HM) contamination. If HMs are present in soils used for agriculture, there is a risk of metal uptake by edible plants with subsequent bioaccumulation in humans and animals and detrimental consequences for their health. Plant productivity can also be negatively affected. Many bacteria have defensive mechanisms for resisting heavy metals and, through various complex processes, can improve plant response to HM stress. Bacteria-plant synergic interactions in the rhizosphere, as a homeostatic ecosystem response to HM disturbance, are common in soil. However, this is hard to achieve in agroecosystems managed with traditional practices, because concentrating on maximizing crop yield does not make it possible to establish rhizosphere interactions. Improving knowledge of the complex interactions mediated by plant exudates and secondary metabolites can lead to nature-based solutions for plant health in HM contaminated soils. This paper reports the main ecotoxicological effects of HMs and the various compounds (including several secondary metabolites) produced by plant-microorganism holobionts for removing, immobilizing and containing toxic elements.

## 1. Introduction

Agroecosystems provide several ecosystem services [1], such as food and raw materials (e.g., wood, biofuels and fibers), which are essential for human life and activity. The surface covered by arable agriculture is about 13% of the global land surface and another 13% is represented by grassland for grazing [2]. The growth in human population requires an increase in food resources and many types of agriculture have been showing a growing trend over the last decade. For example, tree crops have a global extent of about 10 Mha and a ∼20% increase in productivity for many fruit varieties was reported in the decade 2004–2014 [3]. 

Soil is a resource of enormous importance, but a finite and non-renewable one, and several anthropogenic activities are threatening its quality and long-term use, with loss of its key functions. Soil overexploitation by non-sustainable agriculture and over-grazing, contamination by industry and urbanization are the main causes of its deterioration and more than 24% of global land area is estimated to be degraded [2]. Fertile and unpolluted soils are necessary for ensuring healthy crops destined for human and animal use. In particular, soil contamination (e.g., from heavy metals) can seriously hamper soil biodiversity, fertility and crop productivity [4,5] and make agricultural products toxic. Heavy metals (HMs) are among the most common soil contaminants and their presence in concentrations higher than is natural poses a risk because of their toxicity, [6,7,8], bioaccumulation and biomagnification [9].

Microorganisms are a key soil component, ensuring the soil quality and fertility necessary for high-rate production of crops [4,10]. Soil microorganisms, above all *Bacteria* and *Archaea*, represent the majority of soil biomass and are termed “chemical engineers” [4], because they decompose organic matter and make it possible to recycle nutrients through anaerobic and aerobic reactions, involving up to 90% of soil energy flux. Thanks to their small dimensions and fast reproduction capability, prokaryotic cells adapt promptly to environmental changes. They show homeostatic capabilities versus contaminants and can be considered good biological indicators of soil quality [11,12,13,14]. The rhizosphere is a microhabitat, comprising roots and the 1–2 mm soil immediately surrounding them, where there is an intense chemical dialogue between plants and microorganisms [15,16]. In the rhizosphere, plants release root exudates, which promote bacterial population development. The rhizosphere offers a variety of carbon rich micro-habitats, which can be colonized by beneficial bacterial populations using such substrates [3,15].

Microorganisms communicate with plants through chemical messages and develop synergistic actions which influence plant functions and productivity, in both optimal and stress conditions [15,16,17,18]. 

Microorganisms have been found both on and inside plant tissues, but especially at root level [19]. The plant microbiome comprises the rhizosphere, phyllosphere and endosphere [20]. Healthy plants host symbiotic and non-symbiotic rhizo-epiphytic and/or endophytic microorganisms, which do not cause diseases, but support the host nutritionally, by stimulating germination and growth, or helping plants to overcome biotic or abiotic stress. Plants can be considered metaorganisms with close relationships with their associated microorganisms [21]. Indeed, according to the holobiont theory, hosts, such as plants and their microbiome, are symbionts [19,22]. Plant life is closely linked to key microbes, which can influence several aspects of plant ecology, such as growth, germination, biotic and abiotic stress resistance and fitness [19,21,22]. This theory suggests which host-microbiome relationships evolve over time, not just during a single host lifetime, but also through a coevolutionary process, leading to very complex relationships in microorganism-root systems [23]. However, most complex plant-microorganism interactions and chemical dialogues have not so far been understood. Most microorganisms are uncultivable and there are practical difficulties in collecting and separating rhizo-epiphytic and endophytic microorganisms [24]. 

Developing new technologies and nature-based solutions to prevent deterioration of soil and remediation of contaminated sites, while at the same time maintaining soil functions, is a matter of great interest for science and a challenge for the coming decade. In accordance with the One Health Concept, human, animal and environmental wellness and health are tightly related to each other and it is not possible to take an action concerning one of them without considering the others. This approach is of special importance in guaranteeing food safety and sustainable crop production [25,26,27].

The complex and synergistic actions established in the rhizosphere between tree roots and natural underground microbiota make it possible to remove, convert or contain toxic substances in soils, including trace elements [28,29]. Heavy metals (HMs) are among the most widespread soil contaminants worldwide and their presence is reported in 60% of polluted land [30]. The presence of heavy metals at concentrations higher than natural ones poses a risk because of their toxicity [6,7,8], bioaccumulation and biomagnification [9]. These contaminants are a major risk, especially in the most industrialized and populated regions of the earth, endangering human safety and altering ecosystem functions [29,31]. 

If heavy metals are present in soils used for agricultural practices, there is a risk of metal uptake by edible plants, with a subsequent possible bioaccumulation in human and animals and detrimental consequences for their health [31,32]. 

A better knowledge of plant–microorganism interactions is therefore urgently needed to develop correct agronomic management and naturally based solutions, such as phytotechnology for remediation purposes. For this purpose, an ecological approach, taking site-specific biotic and abiotic interactions between plants and microorganisms into consideration, is necessary.

Although, overall, rhizosphere interactions also include fungal mycorrhizae, this review summarizes current knowledge of rhizosphere chemical communication between plant roots and the prokaryotic community associated with them. Particular attention will also be paid to plant and bacteria secondary metabolites.

Central to this discussion is the recent progress made in understanding rhizosphere chemical dialogues between plants and different components of the microbial community and how they can improve plant response to stress by HMs, reducing the effects and toxicity of these chemicals. 

## 2. Heavy Metals

HMs are generally considered those metals and metalloids with an atomic number of at least 20 and a density higher than 5 g/cm^3^ [33]. They are normally present in ecosystems as “trace elements” [29], since many minerals and rocks can contain and release them through erosion and water dissolution. 

Some heavy metals are essential micronutrients for plants, microorganisms and organisms at higher trophic levels (e.g., zinc, iron, copper, nickel). Other HMs (e.g., mercury, cadmium, lead and arsenic) are non-essential elements [33]. Unlike organic substances, they are non-biodegradable. Each metal can become toxic if released in higher concentrations than natural ones, or in the case of chronic exposure [33,34,35]. 

Lead (Pb), Copper (Cu), Cadmium (Cd), Chromium (Cr), Mercury (Hg), Zinc (Zn), Arsenic (As) and Nickel (Ni) are the most common heavy metals found in contaminated soils [33,36,37].

Several anthropogenic activities are responsible for releasing high amounts of heavy metals into natural ecosystems, increasing their concentrations to levels far higher than natural ones and causing, in many cases, serious contamination issues [38,39]. Pollution can be of particular concern when it occurs in agricultural fields, since it can increase the risk of biomagnification and heavy metal uptake, impacting animal and human health [40,41]. Regarding heavy metals (metals and metalloids) in agricultural soils in the EU, Tóth et al. [40] reported that 6.24% of agricultural land shows trace elements (e.g., As, Cd, Cr, Cu, Hg, Pb, Zn, Sb, Co and Ni) exceeding legal limits. In Europe, there are 137,000 km^2^ of contaminated agroecosystems requiring remediation actions. However, there is much more contaminated agricultural land in other parts of the world, where the risk of heavy metal contamination is much more alarming [31,38,42,43,44]. 

Major sources of anthropogenic heavy metal release are mining, industrial activities (especially tannery, smelting and steel mills), atmospheric deposition, sewage from industries and cities, incorrect waste disposal (for example in the case of batteries), fossil fuels and some pesticides and fertilizers [33,36,39,45]. In particular, phosphate fertilizers are a source especially of Cd and can also release Zn, Hg, As, Pb, Cr and U, if they are produced by the acidulation of phosphate rocks naturally containing these metals. Some pesticides can also contain Hg, As and Pb [39,45]. Many pesticides are currently forbidden in some countries, such as the UK [45], while fertilizers in Europe are subjected to regulation in order to limit heavy metal inputs [45,46]. In any case, even if trace element input is limited, their massive use in recent decades has led to HM accumulation in soil [39]. Moreover, crop irrigation with polluted wastewaters can be another heavy metal source [38,45,47].

Heavy metal mobility and bioavailability influence the proportion of metals which can directly interact with living organisms. A part of the total metal concentration in soil is irreversibly linked to or sequestered by the soil matrix and only HMs in a solution are directly available for biota and can be acquired by plant roots [33,35,48]. Zhang et al. [31] reported that heavy metal concentrations found in plant tissue were related to bioavailable metals more than to total ones. Consequently, total heavy metals in soil is not a good parameter for clearly evaluating the possible organism accumulation risk. Many soil properties, such as pH, redox potential, texture, clay content and presence of soil organic matter (SOM), can interact with heavy metals and influence their concentration in a soil solution. In the presence of a low pH, HMs are not specifically adsorbed to soil particles and they are generally more mobile and bioavailable; on the other hand, heavy metals can form stable complexes with SOM, such as humic and fulvic acids [49].

## 3. Heavy Metal Toxicity

Heavy metals can be toxic for biota at all trophic levels, including human. Despite large differences in organization and complexity, HM action mechanisms are similar among organisms, since they act primarily at a cellular level in highly conserved systems. Metals are generally cytotoxic and genotoxic with direct effects on cellular activities [18], which can have several consequences depending on the target organism. Heavy metal characteristics which are responsible for their primary toxicity are:

- high affinity for negatively charged cellular groups, such as sulfhydryls, phosphates and hydroxyls;
- generation of reactive oxygen species (ROS), causing oxidative stress;
- competition with essential ions acquisition;
- disturbance of cellular ion balance and osmotic regulation.

Heavy metals can interfere with normal cellular processes and metabolic functions, causing cellular damage. The affinity for negatively charged groups acts at all trophic levels, causing cell membrane alteration and lipidic peroxidation, with consequences for cell growth and division [41]. Eukaryote membranes of organelles, such as mitochondria, peroxisomes and chloroplasts, can be altered, causing severe damage. For example, in plants, HMs can alter chloroplast inner membrane organization, essential for photosynthesis, potentially leading to a decrease in photosynthetic rates [41,50]. At the same time, heavy metals can bind thiol groups of proteins causing alteration of their structure and leading to protein denaturation and/or loss of functionality [50]. Similarly, metal cations binding to the catalytic sites of enzymes are responsible for direct alteration of their activity. There is great concern about damage to nucleic acids; metal cations can cause the inhibition of transcription and replication and changes in DNA structure, i.e., mutagenic effects, and hinder cell division and cellular cycle [34].

Heavy metals can interfere with cellular redox systems, with the generation of ROS; the disturbance of mitochondrial activity can also cause a lowering in respiration rates in plant tissues. ROS can cause further stress and damage to DNA or other biomolecules, potentially resulting in inhibition of photosynthesis in plants [18,41].

Moreover, heavy metals can interfere with the consumption of essential plant nutrients because of competition with their uptake systems. They can act as antagonists of other ligands of biomolecules and can disturb nutrient translocation systems [37]; a deficiency in micronutrients can have numerous effects, because they are involved in a wide number of biological activities. In different plant species, Cr^+3^ and Cr^+5^ can interfere with uptake of Mg, Mn, Fe, Cu, Zn, but also P and K [37]. Cd, Ni and Pb can replace other ions, such as Zn, in enzymes involved in chlorophyll and other pigment production, lowering photosynthetic rates [41].

Moreover, cellular ionic balance is an issue for both plants and microorganisms, since alteration of cation consumption can generate osmotic problems. In plants, for example, Zn and Pb contamination can contribute to altering the plant water balance [34,41]. It is important to highlight that such effects can also be caused by essential elements if they are in excessive quantities (Table 1), such as in the case of Ni [41].

Damage at cell level is related to that at an organism level. Heavy metal effects on plants are of particular concern, because their stress and toxicity can directly affect crops, decreasing their productivity. Moreover, contamination can be a serious threat for food security in the case of bioaccumulation [35,50]. In fact, heavy metal toxicity can influence plant growth, root elongation and seed germination [50]. It is also reported that heavy metal toxicity can lower plant resistance to other stresses, e.g., pest invasion. Lakshmanan et al. [51] reported that rice plants subjected to arsenic contamination showed higher vulnerability toward rice blast infection.

The sensitivity of plants to heavy metals can be different. There are several species able to survive and grow with a high level of heavy metals; they are termed hyperaccumulating plants and can accumulate up to 100 μg/g of each metal, such as Cd and Cu, and much higher concentrations of other metals [56]. More than 500 taxa are considered hyperaccumulating plants. These species can be very useful for remediation purposes; however, it is not desirable to use them for edible crops in contaminated sites [29,56]. 

Zhang et al. [31] reported concentrations of Pb in rice crops up to 10 times higher than Chinese legislative limits and cases of Cd contamination of wheat, which is one of the most widespread cereal crops worldwide, are of great concern [57]. Unfortunately, heavy metal contamination has been reported in a great number of crops (e.g., brassica, soybean, sugar beet, potatoes and lettuce) all over the world, demonstrating that this problem is here and now and widespread [44].

If heavy metals are accumulated in plants, they can be directly toxic even for animals and humans, through ingestion of contaminated food. Cd poisoning is also reported for tobacco smoking [35] and HM skin contact can cause irritation and allergic reactions in humans. Several illnesses are reported to be linked to heavy metal poisoning. In fact, many heavy metals, such as Hg, Pb and As, are known to be toxic for the renal, cardiovascular, gastrointestinal and nervous systems. Lead poisoning, for example, can cause headache, mental confusion and disorientation [50]. Permanent damage can occur after long-term exposure and some metals are thought to be carcinogenic [18].

## 4. Heavy Metal Bacterial Resistance

Long-term heavy metal contamination in soil is a selection pressure which can promote bacterial species able to develop HM resistance [14,58]. Bacteria can help plants to resist stress and improve plant growth and productivity. This is possible thanks to bacterial transformation of HMs into less toxic forms and alteration of their availability [17,59] (Table 2). Understanding microorganism resistance mechanisms and their relationships with plants can make it possible to develop more efficient and specific technologies for heavy metal bioremediation to apply to crops [60]. These resistance mechanisms, involving a chemical dialogue between bacteria and plant, include secondary metabolites and various complex processes. 

Several bacterial species can actively transport toxic metals outside the cell, e.g., through expression of the ATPase efflux mechanism and ion pumps. The genes for such bioexclusion mechanisms are generally plasmid encoded [36,50,61,62].

Bacteria can transform heavy metals through redox reaction and methylation [36]. Changes in metal redox state can alter metals’ solubility in soil, influencing their toxicity. Transformation of metals into less toxic or less mobile forms can reduce phytotoxic effects and be advantageous for plants [17,52,63]. Moreover, methylation of HMs can also lead to forms with increased solubility but lower toxicity and can sometimes produce more volatile forms, such as in the case of Hg [17] (Ma et al., 2016) and As [64,65,66].

Biosorption is a widespread metal resistance mechanism. It is a metabolism-independent and passive process, which can also involve death cells [67,68]. Biosorption consists in attachment of metal cations to the cellular surface, because it is generally negatively charged for the presence of several anionic functional groups. Exopolysaccharides (EPS) and humic acids can also be released from the cell and help HM acquisition [36,60,69].

Biosorption contributes to metal immobilization, lower toxicity and the blocking of metals from entering cells (including plant cells) [70]. Moreover, it helps metal sequestration through chelation or subsequent bioaccumulation [63,71]. 

Bioaccumulation is an active and metabolism-dependent mechanism that makes it possible for metals to enter a cell. It requires cell membrane carriers and pumps [17]; the production of metal-binding proteins inside cells allows sequestration and immobilization of high concentrations of metals, thus reducing stress [58,72,73]. Metallothioneins are common binding proteins involved in metal immobilization; their expression is induced directly in the case of exposure to heavy metals in a stress condition [36,73,74].

Moreover, production of chelating compounds released outside cells can also contribute to heavy metal immobilization, protecting microbes and plants from HM acquisition and toxicity [75,76]. For example, biosurfactants are natural chelating agents, with a variable composition; they are secondary metabolites, which include glycolipids, polysaccharide, lipoprotein and mycolic acids. Metallophores and low molecular weight organic acids (e.g., formic, citric, oxalic and acetic acids) produced by both microbes and plants can bind metal cations [73,77]. However, most microbial chelating agents studied are siderophores, which are involved in Fe^+3^ acquisition and in increasing its bioavailability. Fe^+3^ is an essential micronutrient for plants and microbial siderophores can have a higher affinity for iron than phytosiderophores [17]. Siderophore-producing microorganisms can help plant Fe^+3^ acquisition in iron deficient soil, helping plant growth and productivity [17,36].

Different bacterial resistance mechanisms can all contribute to metal immobilization in soil, which can be desirable in the case of HMs present in soil used for agricultural purposes. Biosorption, bioaccumulation or modification of a chemical state can cause an overall lowering in metal availability [52,73] and a consequent lower HM concentration in plant tissues [53]. Han et al. [53] isolated a metal resistant *Enterobacter bugandensis* TJ6 bacterial strain from the rhizosphere of a metal-contaminated lettuce crop. *E*. *bugandensis* TJ6 was able to bioadsorb, bio-precipitate and bioaccumulate Cd, leading to Cd immobilization and to a lowering in metal concentration in wheat tissues (grains, straw, roots). The bacterial strain was also able to enhance urease activity and produce secondary metabolites, such as indoleacetic acid, promoting crop growth in a heavy metal presence. The use of this bacterium for bioaugmentation purposes could be a promising technology for enhancing crop safety.

## 5. Plant–Bacteria Interactions in Rhizosphere and Defense from Heavy Metal Stress

Microorganisms have an important role in pollutant detoxification and heavy metal plant stress resistance. Free-living microorganisms, as well as organisms more strictly associated with roots and endosymbionts colonizing plant inner tissues [3,14,15], make it possible to build complex communities promoting plant colonization by beneficial species, which increase vegetal growth, discourage pathogens and can promote heavy metal removal [29,102].

Plants contribute to the assemblage of their own rhizobiome [3,16] and some plant species can have their specific microbial community [102], which can, in turn, change during their growing stage and in different root regions [16]. Plants grown in polluted soils or in stress conditions, such as with a heavy metal presence, can establish a special rhizobiome useful for their resistance [18,103]. For example, Sun et al. [42] found that rhizosphere communities in HM contaminated soils were crop specific and specific metal–microbe interactions were found for rice, soybean or corn. 

The presence of plants in contaminated soils promotes changes in microbial communities, increasing their biomass, diversity and activity and promoting bacterial heavy metal detoxification [104,105,106,107]. Root exudates can act as electron donors and many compounds can be used by metal-reducing bacteria to detoxify heavy metals [108].

The positive “rhizosphere effect” can also influence microbial communities in the bulk soil not far from roots, causing a general improvement in soil quality [14]. 

Hyperaccumulative plants can acquire heavy metals, thanks to exudates which enhance soil pH, promoting metal mobilization and bacterial detoxification [109]. Hyperaccumulative ecotypes of plants, such as *Sedum alfredii*, show different microbial community compositions compared to non-accumulative ones. The rhizospheres of such ecotypes also show higher enzymatic activity and a decrease in soil HM content [106]. 

In particular, bacteria with a beneficial activity for plants are often defined as plant growth-promoting rhizobacteria (PGPR) [18]. Through the production of secondary metabolites (e.g., siderophores, 3-indoleacetic acid: IAA, 1-aminocyclopropane-1-carboxylic acid: ACC), deaminase activity and a capability to solubilize phosphates [66,110], PGPR are directly involved in metal detoxification. Several metal tolerant PGPR are involved in phytoremediation, because they alleviate both plant metal toxicity and stress and can alter metal mobility and increase plant growth [17,59,66]. PGPR includes a high number of genera that can be associated with roots, such as *Bacillus, Pseudomonas, Enterobacter, Acinetobacter, Burkholderia, Arthrobacter, Paenibacillus* [18], *Agrobacterium, Lysinibacillus* and *Flavobacterium* [91] and new genera are continuously being found. 

### 5.1. Heavy Metals and Secondary Metabolites

The sophisticated mechanisms of chemical communication between plants and microorganisms involve a great diversity of exudates (sugars, amino acids, nucleotides, phosphate groups, peptides), many of which are secondary metabolites [16,17,111]. Because their production involves a significant carbon cost for plants, the advantages are presumably substantial [15,102]. 

Plant secondary metabolites are small molecule metabolism products, which are non-essential for the survival of the organism and many of them are defensive compounds. They include flavonoids, phytohormones and terpenoids. Secondary metabolites are involved in plant protection against herbivores, bacteria, fungi, viruses and even competing plants. In addition, some plants use secondary metabolites as signals for communication between them and symbiotic microorganisms and for attracting pollinators and seed dispersers [112].

Similar to plants, bacterial secondary metabolites are not necessary for or directly related to cell growth and reproduction, but they can be involved in ecological interactions with other microorganisms and plants in the rhizosphere. Bacterial secondary metabolites are generally part of a few compound classes originating from defined biosynthesis pathways; however, in each pathway, variations in enzymatic activity, specific assembly and modification can lead to a great diversity in molecules for each compound class. Bacterial secondary metabolites comprise antimicrobial peptides, lipopeptides, phytohormones or their precursor, acyl-homoserine lactone, nitrogen compounds, siderophores, metallothioneins [113], exopolisaccarides and volatile organic compounds [114].

The composition of root exudates can be different, depending on plant species [16,115], growth phases [16,116], exposure of a plant to stress conditions [117] and sometimes differences in plants of the same species [118]. However, in many cases, the molecular structure of these compounds is still not known [16,111]. For example, in the plant *Arabidopsis thaliana*, more than 500 compounds have been detected at different growth stages [119] and with a natural intraspecific variability [118]. Zhalnina et al. [116] found that different growth stages of *Avena barbata* were characterized by different exudate compositions. At the same time, different bacterial populations related to the rhizosphere were found at different plant growth stages, because bacterial populations were able to optimize utilization of root exudates at each plant stage, adjusting their gene expression.

The maximum diversity in exudate compositions has been generally found during maximum vegetative growth; this was also related to maximum expression of bacterial genes related to nutrient (N and P composes) acquisition. Changes in exudation pathways during different plant growth stages are therefore essential for recruiting bacterial populations which can support plant growth [119]. Plant and rhizobacteria genomes, strictly linked in a coevolutionary process, can be defined as a hologenome [104,116].

#### 5.1.1. Heavy Metals and Flavonoids

Flavonoids are plant secondary metabolites released by roots. Flavonoids are known to act as chemoattractants, which induce the expression of bacterial nod genes and production of lipo-chitooligosaccharides (LCO), essential in nodules formation in roots. Flavonoids have plant bacteria specificity; in fact, different compounds can attract different bacterial species, making possible colonization of a specific guest [17,102]. Nitrogen fixing bacteria occurring in root nodules are PGPR investigated in the search for new species to be used in bioremediation [120], since nitrogen fixation can stimulate plant growth in HM contaminated soil [121,122]. Association of rhizobia with legume roots can increase phytostabilization of HMs (Cd, Cu, Pb), reduce metal translocation to aerial parts and increase plant nitrogen content and growth [121]. Nitrogen fixing capacity is crucial for plant adaptation to soil that is extremely poor in nutrients and strongly contaminated, such as mine trailing soil [123].

Plants produce flavonoids for alleviating metal stress, enhancing antioxidant activity [124,125]; their release in soil can be considered a plant resistance mechanism. In particular, flavonoids can chelate metals (e.g., Fe, Cu, Ni, Zn) [124] and can contrast ROS inside plant cells [125]. 

Flavonoids are also essential for chemical signaling with mycorrhizal fungi and rhizobacteria [126,127]. They act as chemoattractants and can directly stimulate bacterial gene expression [126,128]. Flavonoids include a wide variety of chemicals and can have an antimicrobial action against bacterial and fungal root pathogens [127,129,130] and support crop stress resistance [129]. 

Flavonoids can significantly stimulate the dehydrogenase and protease activity of rhizobacterial communities, involved, respectively, in SOM oxidation and nitrogen cycling, although the effect on bacterial abundance is still not clear [131].

The role of PGPR interaction with flavonoids in alleviating plant metal stress is not completely clear. For example, Khanna et al. [132] found that inoculation of *Solanum lycopersicum* seeds subjected to Cd stress with a PGPR strain strongly increased plant synthesis of flavonoids and other phenolic compounds, enhancing antioxidant activity and alleviating metal toxicity. However, Ullah et al. [121] reported that inoculation with two endophyte PGPR strains (*Serratia* sp. IU01; *Enterobacter* sp. IU02) in the hyperaccumulating plant *Solanum nigrum* reduced plant production of several enzymes and secondary metabolites with an antioxidant activity, including flavonoids. This result could be attributed to bacterial production of exogenous antioxidants, leading to a reduction in plant-antioxidant production and, in any case, plant stress. This phenomenon has been found for production and activity of super-oxide dismutase (SOD) and it is reasonable to hypothesize that this can also happen for other compounds. Moreover, the same author [121] described that the PGPR strains applied enhanced Cd accumulation in roots (i.e., phytostabilization) and restored plant growth initially affected by HMs. 

The toxic effect of HMs on natural soil microbial communities can be reduced or not be evident in soils rich in organic carbon and, in particular, in humic acids, which can chelate them [133]. Rhizosphere chemical signaling can be influenced by various soil abiotic factors (e.g., organic carbon content, temperature, soil texture, water availability and aeration), but this aspect has not been comprehensively investigated so far [111,123]. A recent study highlighted that soil organic carbon can cause an attenuation of flavonoid signaling, owing to flavonoid complexation with SOM phenolic compounds, through a metal-mediated binding [123].

#### 5.1.2. Heavy Metals and Quorum Sensing

Some bacteria produce secondary metabolites, such as acyl-homoserine lactone (acyl-HSL), which is considered an autoinducer signal. Autoinducer production in some bacterial strains can directly activate genic pathways involved in metal regulation and resistance; in such cases, a single-cell gene response can be a consequence of a population response [134,135]. Quorum sensing (QS) is a recognized communication mechanism used by bacteria to guide coordinated responses in bacterial populations [17]. QS makes possible root adhesion and colonization, biofilm formation, mobility, gene expression and stress responses [111]. QS is regulated by bacterial signals, especially acyl-homoserine lactone (acyl-HSL) produced by Gram-negative bacteria [136]. Acyl-HSLs are produced by singular cells and then released among a population, stimulating an overall response when they reach a specific concentration [17,136]. QS is involved in interspecific communication, mediating cooperation and competition in bacterial populations and mutualistic and positive interactions of bacterial species in the rhizosphere [137].

In fact, bacterial mutants in QS pathways can show reduced plant growth promoting ability and a lower success in root colonization [136].

Acyl-HSLs can promote plant growth [138] and stimulate plant defense-genes and resistance to biotic stress [139] and abiotic stress, such as salt stress [140]. Many studies have shown that treating seeds with acyl-HSLs has beneficial effects on both crops and root bacteria affected by HM stress [140,141]. However, the action mechanism of acyl-HSLs involved in mitigating stress in plants needs to be clarified [139].

QS can also be involved in exopolysaccharide (EPS) and biofilm production [142]. EPS are macromolecules with a polysaccharidic central structure and variable side chains [76]. They are released outside bacterial cells and are the main component in the biofilm matrix [69]. EPS are involved in bacterial–plant interactions, such as host specificity, symbiosis and plant defense toward stress; moreover, EPS production is considered a PGP trait [18].

EPS can be involved in bacterial metal resistance sequestering HMs outside cells through anionic functional groups [70,82]. 

Several studies report PGPR species capable of biofilm formation and EPS production, which promote plant growth, alleviating heavy metal toxicity and decreasing HM plant uptake. It is reasonable to think that EPS are involved in plant tolerance to HMs, although no direct link between EPS production and plant HM resistance has been demonstrated [70,71,82].

Biofilm and EPS are widely used for bioremediation of several contaminants and their action in HM removal has been thoroughly investigated [69,76]. Combining current knowledge of EPS with phytoremediation could improve bioremediation effectiveness.

#### 5.1.3. Heavy Metals and Phytohormones

Phytohormones are involved in plant growth and stress responses and can take part in rhizosphere communication [111]. Phytohormones production and release by roots can be essential for rhizobiome assemblage, since they can be used as signal molecules and carbon and nitrogen sources by microorganisms [143]. Lu et al. [144] studied 17 phytohormones in plant-rhizosphere-bulk soil systems, highlighting three different distribution patterns, depending on the production and degradation pathway of each phytohormone by plants and/or bacteria. Moreover, the sterilization of the rhizosphere microbial community significantly influenced phytohormone production, leading in many cases to a reduction in phytohormone concentration. A strong role and influence of the bacterial community in plant hormone pathways has also been recognized [144]. Plant bacterial symbionts can both produce exogenous phytohormones and modulate endogenous plant hormone production, demonstrating a crosstalk between plants and bacteria [145]. Indeed, in root exudates, there are some molecules that can be used by bacteria as a precursor for synthetizing phytohormones. For example, tryptophane is a precursor of 3-indoleacetic acid and can be found in high concentrations in root tips; similarly, amminocyclopropane-1-carboxilic acid is the precursor of ethylene and is released in exudates [102,146]. In other cases, rhizobacteria can produce the precursors of phytohormones, which are converted in the final molecule into plant tissues [147]. Several bacterial strains (e.g., *Enterobacter* sp. SA187, *Azospirillum* spp.) can directly alter plant gene expression, influencing phytohormone metabolic pathways [147,148]. The main phytohormones reported to be influenced by bacterial activity are gibberellins (GA), abscisic acid (ABA), jasmonic acid (JA) [145,149], salicylic acid (SA) [145,150], 3-indoleacetic acid (IAA) and cytokinin [149]. 

The bacterial phytohormones production capacity is well known and it is widely distributed among bacteria plant symbionts [151]; in fact, phytohormone production, in particular, of IAA, is another trait used for identifying plant growth-promoting rhizobacteria (PGPR) species. 

Phytohormones are specific for a plant life cycle; however, bacteria can take part in their regulation and synthesis. There are sophisticated relationships in the rhizosphere presumably deriving from a precise coevolutionary process [152].

Phytohormone production capacity can be an efficient system for promoting plant resistance to biotic and abiotic stresses, while, at the same time, enhancing plant growth [145,148,149,150]. 

The hormonal crosstalk between plants and bacteria is also involved in responses to HMs. Several studies show rhizobacteria able to decrease the stress effect of HMs and influence different phytohormones [153], for example, by restoring their production when hampered by metals, or by lowering levels of overexpressed hormones. Bilal et al. [154] demonstrated that co-inoculation of soybeans with endophytic bacteria and fungi (able to produce phytohormones) significantly decreased the stress effect from Al and Zn contamination. The co-inoculation decreased HM plant uptake, directly influencing its metal-transporter genes. Moreover, microorganisms promoted plant growth, favoring plant gibberellin production and regulating abscisic acid content. Moreover, Qadir et al. [155] showed that *Helianthus annuus* seeds grown in the presence of Cr showed a significant decrease in accumulation of endogenous IAA. However, the inoculation of seeds with a PGPR strain (*Staphylococcus arlettae*) positively influenced seed IAA production, increasing sunflower response to heavy metal stress.

In fact, rhizobacteria can modulate plant phytohormones involved in stress responses through sophisticated mechanisms. Ravanbakhsh et al. [156] demonstrated that a *Pseudomonas putida* strain decreased ethylene (ET) production in several plant species, through the expression of 1-aminocyclopropane-1-carboxylate deaminase (ACC-deaminase), an enzyme involved in the degradation of the ethylene precursor. Plants inoculated with ACC-deaminase producing bacteria showed a significantly lowered cadmium uptake, probably because ET is involved in the expression of metal transporters, so that a reduction in ET can reduce metal uptake. In this case a reduction in ethylene led to a reduction in plant growth; consequently, microorganisms helped plants in metal resistance, but did not act as PGPR.

It is interesting to note that exogenous hormones applied to plants can show different effects. Chen et al. [157] compared the effect on *Sedum alfredii* growth and Cd uptake of only exogenous IAA and the effect of adding IAA producing endophytic bacteria (*Pseudmonas fluorescens*). The results showed that IAA could increase shoot biomass, enhancing plant growth, but did not influence Cd accumulation. Conversely, the bacterial strain influenced plant growth more strongly, enhancing both root and shoot biomass and increasing plant Cd phytoextraction capacity. The effect on a plant of a rhizobacterial strain therefore depends partially on its IAA producing ability and much more on other bacterial features. 

Most studies have focused on the activity of one or a few phytohormones, but hormonal crosstalk could be much more complex than expected. Wu et al. [158] demonstrated that the inoculation of the hyperaccumulating plant *Sedum alfredii* with a PGPR strain caused a strong increase in lateral root formation and Cd acquisition, enhancing phytoremediation efficiency. Transcriptome analysis of the inoculated plant showed an up-regulation of 146 extra genes involved in hormonal balance, compared to a control. The presence of rhizobacteria promoted a cooperation of more hormones. Such effects more likely represent what really happens in the rhizosphere.

#### 5.1.4. Heavy Metals, Siderophores and Metallothioneins 

Siderophores are secondary metabolites produced by both bacteria and plants, in the latter case termed phytosiderophores. In both bacteria and plants, they can enhance uptake of iron, an essential micronutrient [159]. To increase their wellness, some plant species select the microbial community, through root exudates, with the best siderophore-producing capacity [160]. Most bacteria producing siderophores in the rhizosphere are free living ones, because they are directly in contact with cations [161,162]. Different plant species have microbial communities with different siderophore producing capacities. For example, bacterial strains with a high siderophore production capacity were isolated from roots of hyperaccumulating plants [162].

Siderophores help plants not only in providing essential nutrients (e.g., iron) in stress conditions, but also in helping them to detoxify soil of heavy metals. Siderophores can bind several metals, such as Cr^+3^, Al^+3^, Cu^+2^, Pb^+2^, Zn^+2^ and Cd^+2^. In some cases, heavy metals are sequestered outside cells [157,159,161,163]. In other cases, such as in the rhizospheres of hyperaccumulating plants, siderophores can enhance metal mobilization, increasing plant uptake and helping in phytoextraction [162,164,165]. Yu et al. [166] demonstrated that glucose and lead stimulated siderophore production in the bacterial strain *Bacillus* sp. PZ-1; the subsequent soil bioaugmentation with this strain, increased lead acquisition from *Brassica juncea*, presumably because of the bacterial siderophore production.

However, as above mentioned, even in the absence of plants, metals stimulate siderophore production in several bacteria, indicating their direct capacity to resist metal stress [161,167].

Another class of secondary metabolites and chelating compounds involved in heavy metal detoxification are metallothioneins, which are produced by a wide range of organisms, including plants and bacteria. They are small cysteine-rich proteins, have a high metal-binding capacity and are, in fact, involved in metal detoxification and storage [168]. Metallothionein production is stimulated by heavy metals (e.g., Cd^+2^, Zn^+2^, Pb^+2^, Cu, Cd); metallothioneins chelate hazardous metals and sequestrate them inside cells [168,169]. In plants, metallothioneins are also involved in translocation and homeostasis of essential cations, such as Zn and Cu [169].

Numerous bacterial metallothioneins have been identified, together with their genetic pathways in several bacterial strains [168]. A large difference in aminoacidic sequences suggests a high diversity in metallothionein evolution pathways [170]. However, many metallothioneins are still unknown and further investigations will unveil other molecules. Because, bacterial metallothioneins can increase metal uptake, reducing their toxicity, they can be an efficient solution for improving heavy metal bioremediation [168,170].

Plant metallothionein producing capacity is spread among several species. Metallothioneins not only increase metal tolerance and uptake, but also help plant growth [169]. In a similar way to bacteria, plant metallothioneins are widely studied and they may represent a promising strategy for enhancing plant resistance in heavy metal contaminated soil, particularly for increasing the efficiency of hyperaccumulating plants [171,172,173].

Finally, there have been several studies focused on metallothioneins, using genetically engineered organisms to test their efficiency [171,172], obtain metal resistant plant species [173], or investigate rhizobacteria–plant symbionts in contaminated soil [174]. However, data on metallothionein plant–bacteria interactions in natural ecosystems are scarce and mechanisms regulating their production still need to be clarified. 

## 6. Conclusions

The chemical dialogue in the rhizosphere is the result of complex mechanisms due to intra and interspecific interactions mediated by a wide variety of molecules, many of them secondary metabolites. Thanks to these interactions, plants and their associated root microbiomes are able to respond to heavy metal stress.

A better understanding of root exudation pathways and especially of the plant and bacterial genes involved in different growth stages and stress responses could be useful for future application in crop management. Inoculating crops with autochthonous bacterial species or secondary metabolites able to help plants in stress resistance could be a promising nature-based solution, which merits being better investigated.

## Figures and Tables

**Table 1:** Heavy metals (HMs), which are micro-essential nutrients for plants but toxic in excessive concentrations. The roles and toxicity of zinc, copper, iron and nickel are reported. The legal limits refer to agricultural soils in accordance with Italian Decree 46/2019. Currently, at the EU level, there is no Directive on soil.

Essential HMs for Plants	Role	Toxicity	Legal Limits mg kg^−1^
Zn^+2^	Cofactor in many enzymes, present in protein–DNA domain interaction (zinc finger proteins); role in plant defense response; response to oxidative stress [52].	It competes with other essential ion adsorptions (Fe^+2^; Mn^+2^, Mg^+2^), it can substitute Mg^+2^ in chlorophyll inhibiting photosynthesis [53].	300
Cu^+^/^+2^	Cofactor of many enzymes necessary in electron transport chain; involved in iron mobilization and in cell wall metabolism; it has a role in plant stress responses [54].	It substitutes Mg^+2^ in chlorophyll, inhibiting photosynthesis; it can cause malfunctioning of photosystems (PSI and PSII); it can cause oxidative stress at higher concentrations and alter root morphology and biomass [53].	200
Fe^+2^/^+3^	Essential in electron transport chain; cofactor of many enzymes; involved in photosynthesis and chlorophyll synthesis [22].	It can cause severe oxidative stress and ROS generation; Fe^2+^ can be responsible for photosystem damage and inhibition of photosynthesis; Fe^+2^ e Fe^+3^ can interact with transport systems for other essential elements [53].	--
Ni^+2^	Necessary for plant growth at low concentration; involved in enzymatic functions necessary for plant redox state maintenance; involved in nitrogen metabolism [55].	It can cause inhibition of growth and biomass accumulation; it can interfere with water and nutrient acquisition; it can cause lipid peroxidation and interfere with pigment production [55].	120

**Table 2:** Bacterial species able to resist HMs through various mechanisms (detoxification, mobilization, immobilization and metal transformation). EPS: exopolysaccharide.

	Bacteria (Genera, Species, or Strain)	Metal and Mechanism of Action	References
**Alpha-Proteobacteria**	*Agrobacterium sp.*	It grows up to 8000 mg/L of As^+5^ and 80 mg/L of As^+3^.	[66]
	*Ochrobactrum sp. GDOS*	It bioadsorbs Cd^+2^.	[78,79]
	*Rhizobium radiobacter F2* *(Agrobacterium tumefaciens)*	It can produce EPSs to bioadsorb Pb^+2^ and Zn^+2^.	[76]
	*Rhizobium viscosum* *(Arthrobacter viscosus)*	It bioadsorbs Cr^+5^ on live and dead cells and reduces it to Cr^+3^ in an aqueous solution.	[68,79]
	*Rhodobacter capsulatus*	It bioadsorbs Zn^+2^.	[78,80]
	*Rhodobacter sphaeroides*	It bioadsorbs Ni^+2^.	[17,81]
	*Rhodopseudomonas palustris*	It has plasmid genes for As^+3^ methylation and resistance; it can increase arsenic volatility.	[64,65]
	*Sinorhizobium meliloti*	It produces EPS to resist As^+3^ and Hg^+2^; it has an efflux pump to exclude As^+3^.	[76,82]
**Beta-Proteobacteria**	*Comamonas testosteroni S44*	It resists zinc (chromosomal gene); it also has plasmid genes encoding 9 active Zn^+2^ transporters, used vs Cd^+2^ and Pb^+2^ too.	[36,62]
	*Cupriavidus taiwanensis E324*	It resists and bioadsorbs Cd^+2^ and Zn^+2^.	[79,83]
	*Herminiimonas arsenicoxydans*	It oxidates As^+3^ and immobilizes it through EPS production; it shows chemiotaxis vs As^+3^; it has a bacterial genome encoding for efflux pump for several metals.	[76,84]
	*Ralstonia metallidurans* *(Cupriavidus metallidurans)*	It resists Pb^+2^; it has chromosomal and plasmid genes which maintain low Pb intracellular concentration.	[50,61]
	*Thiomonas sp. CB2*	It oxidates As^+3^ to As^+5^ and produces biofilm as a As^+3^ stress response.	[76,85]
**Gamma-Proteobacteria**	*Aeromonas sp. CA1*	It resists/grows in presence of arsenic and can reduce As^+5^ to As^+3^.	[36,86]
	*Acinetobacter sp. FM4*	It can bioadsorb Cr^+5^, Cr^+3^, Cd^+2^, Cu^+2^, Ni^+2^ and Hg^+2^.	[50,87]
	*Acinetobacter junii L. Pb1*	It bioadsorbs Pb^+2^ through exopolysaccharide production.	[50]
	*Azotobacter vinelandii*	It produces metallophores to chelate iron and molybdenum.	[17,75]
	*Enterobacter cloacae*	It can bioaccumulate chromium.	[88]
	*Klebsiella planticola*	It precipitates cadmium forming CdS.	[17,89]
**Gamma-Proteobacteria**	*Providencia vermicola SJ2A*	It bioaccumulates lead; it has plasmid genes encoding formetallothioneins production.	[74,90]
	*Pseudomonas aeruginosa*	It sequestrates lead inside cells with production of metallothioneins.	[50]
	*Pseudomonas aeruginosa B237*	It resists and bioadsorbs Cd^+2^ and Zn^+2^.	[79,83]
	*Pseudomonas fluorescens*	It sequestrates lead inside cells with metallothionein production.	[50]
	*Pseudomonas fluorescens RhzP-43, RhzP-44*	It resists copper and zinc up to 50–100 µg/mL.	[91]
	*Pseudomonas putida*	It oxidates As^+3^ to As^+5^ thanks to plasmid genes; it reduces cadmium mobility with EPS production.	[36,76,92,93]
	*Pseudomonas veronii*	It can bioadsorb Cd^+2^, Cu^+2^ e Zn^+2^.	[52,94]
	*Stenotrophomonas maltophilia Rhz-S17*	It grows up to 8000 mg/L of As^+5^ and 170 mg/L of As^+3^.	[66]
	*Stenotrophomonas maltophilia Rhz-S31*	It grows up to 8000 mg/L of As^+5^ and 165 mg/L of As^+3^.	[66]
	*Stenotrophomonas malthophilia/rhizophila RhzS-31*	It resists copper and zinc (up to 50–100 µg/mL) .	[91]
**Actinobacteria**	*Cellulosimicrobium funkei AR8*	It reduces Cr^+6^ to Cr^+3^ and immobilizes chromium on cellular surfaces and bioaccumulates it in cytosol.	[63]
	*Micrococcus luteus DE2008*	It resists and bioadsorbs Pb^+2^ up to 1965 mg/g and Cu^+2^ up to 408 mg/g.	[50,95]
	*Tsukamurella paurometabola A155*	It resists and bioadsorbs Zn^+2^.	[79,83]
**Firmicutes**	*Bacillus sp. PZ-1*	High resistance to Pb^+2^; it can bioadsorb Pb^+2^ and also resist Cu^+2^, Zn^+2^, Cu^+2^, Ni^+2^.	[79,96]
	*Bacillus cereus*	It resists lead withmetallothioneins production.	[50,72]
	*Bacillus cereus RC-1*	It bioadsorbs Cd^+2^ on live and dead cells and bioaccumulates small quantities.	[52,67]
	*Bacillus cereus XMCr-6*	It bioadsorbs Cr^+6^ and reduces it to Cr^+3^.	[52,97]
	*Bacillus sphaericus*	It bioadsorbs chromium and resists arsenic, mercury, iron and cobalt.	[17,98]
	*Bacillus subtilis RhzB-45*	It resists copper and zinc, up to 50–100 µg/mL.	[91]
	*Bacillus thuringiensis 016*	It bioadsorbs Pb^+2^ and precipitates it on cellular surfaces.	[50,99]
	*Exiguobacterium sp.WK6*	It resists and grows in presence of arsenic; it can reduce As^+5^ to As^+3^.	[36,86]
	*Lysinibacillus sp. RhzL-42*	It resists copper and zinc up to 50–100 µg/mL.	[91]
	*Lysinibacillus sphaericus/* *fusiformis RhzL-41*	It resists copper and zinc up to 50–100 µg/mL.	[91]
	*Sporosarcina ginsengisoli*	It resists As^+3^ and reduces its bioavailability forming calcite precipitation.	[100]
	*Staphylococcus epidermidis*	It produces biofilm and removes Cr^+5^ from aqueous solutions.	[79,101]
